# Metabolomic profiling of varicocele-induced male infertility: insights from spermatic vein blood analysis

**DOI:** 10.3389/fendo.2025.1682362

**Published:** 2026-01-07

**Authors:** Xiyi Wei, Zijie Yu, Shuai Wang, Da Zhong, Yichun Wang, Xi Zhang, Wenchuan Shao, Xinghan Yan, Chao Qin, Ninghong Song

**Affiliations:** 1 Department of Urology, The Affiliated Cancer Hospital of Nanjing Medical University & Jiangsu Cancer Hospital & Jiangsu Institute of Cancer Research, Nanjing, China; 2 Department of Urology, The First Affiliated Hospital of Nanjing Medical University, Nanjing, China

**Keywords:** varicocele, male infertility, metabolomics, oxidative stress, aspartate

## Abstract

**Objective:**

Varicocele (VC) is a leading cause of male infertility, but its pathophysiological mechanisms remain inadequately defined. This study aimed to characterize metabolic alterations in spermatic vein blood of varicocele patients with/without infertility (VI/VF groups) compared to healthy donors.

**Methods:**

We collected spermatic vein blood samples from VC patients and peripheral blood samples from healthy donors. VC patients scheduled for surgery were categorized into VF (without infertility) group or VI (with infertility) group based on fertility status, with healthy donors as the negative control (NC) group. Metabolic profiling of spermatic vein blood from VC patients and peripheral blood from healthy donors was performed using liquid chromatography-mass spectrometry (LC-MS). Semen parameters and ultrasound findings were recorded. Principal component analysis (PCA) and orthogonal partial least squares discriminant analysis (OPLS-DA) were employed to identify metabolic differences, followed by pathway enrichment analysis.

**Results:**

A total of 40 VC patients and 35 healthy donors were included in this study. Semen quality in the VI group was significantly compromised compared to VF and NC groups (*P* < 0.001). Metabolic profiling revealed profound alterations in varicose spermatic veins of VC groups, including upregulated steroid hormone biosynthesis, disrupted amino acid and energy metabolism, and elevated reactive oxygen species (ROS) production compared to healthy donors. Differences between VI and VF groups were primarily localized to the alanine, aspartate, and glutamate pathway, with γ-aminobutyric acid, succinic acid, and aspartate significantly upregulated in VI groups (*P* < 0.05). Among renal and adrenal metabolites, only calcitroic acid exhibited differential expression (*P* < 0.01), while oxidative stress markers, including N-acetylcysteine and acylcarnitines (*P* < 0.05), showed significant variation.

**Conclusion:**

Untargeted metabolomics highlighted D-aspartic acid overexpression as a potential contributor to infertility in VC patients. This study provided only limited support for the retrograde reflux theory of renal and adrenal metabolites, while offering additional evidence consistent with the oxidative stress hypothesis. Oxidative stress-related metabolites may represent potential candidate diagnostic and therapeutic targets for VC-induced infertility.

## Introduction

Varicocele (VC) refers to the abnormal dilation, twisting, and thickening of the pampiniform venous plexus within the scrotum, which can lead to testicular damage and decreased sperm quality, closely associated with male infertility (1, 2). VC occurs in approximately 15% of males and is associated with 35-44% of primary male infertility cases, being found in 45-81% of cases of secondary male infertility (2). VC, deemed as the most prevalent remediable cause of male infertility, can give rise to either primary or secondary infertility in men. Nevertheless, despite the presence of varicocele, some individuals exhibit fertility even without intervention. In infertile men with clinically palpable varicoceles and abnormal semen analyses, varicocele repair may provide significant clinical benefit. However, treatment response varies, with a subset of patients demonstrating minimal improvement in reproductive parameters (3, 4).

The pathophysiological mechanisms underlying male infertility caused by varicocele remain to be fully elucidated. Varicocele can induce a series of abnormalities such as decreased serum testosterone levels, scrotal discomfort, testicular dysfunction, and diminished sperm quality. The current mainstream viewpoint suggests that the elevated scrotal temperature may be the primary mechanism through which varicocele affects endocrine function and sperm production, as both are sensitive to temperature changes (5, 6). Another potential mechanism involves the reflux of adrenal and renal metabolites, supported by anatomical radiological studies (7, 8). Additionally, increased venous reflux leading to elevated intravenous pressure within the spermatic cord may also contribute to the pathology of varicocele (9). These pathological effects include hypoxia, increased testicular temperature, altered testicular blood flow and venous pressure, stasis, inflammation, reduced levels of gonadotropins and androgens, oxidative stress (OS), and cellular apoptosis (10).

Metabolomics primarily investigates the comprehensive metabolic responses of living organisms to exogenous stimuli, environmental changes, or genetic modifications, and delineates the holistic dynamics of metabolic profiles (11). This approach has been applied to research on male fertility to identify metabolic pathways and biomarkers potentially associated with male infertility, thereby guiding the development of precise therapeutic strategies for male infertility (12). However, there is limited research on metabolomics of varicocele, with most studies focusing on seminal plasma or semen samples as the research subjects (13–15). The predominant focus of these investigations lies in disease characterization and the establishment of diagnostic frameworks, yet the precise pathological elucidation of the condition remains elusive.

Current research on varicocele pathophysiology has identified significant biochemical alterations in spermatic venous blood. Postoperative analyses consistently suggest elevated testosterone derivatives (total testosterone, free testosterone, and dihydrotestosterone) and estradiol levels in the internal spermatic vein compared to peripheral circulation, suggesting both impaired steroidogenesis and hormonal distribution abnormalities (16). The well-characterized phenomenon of excessive nitric oxide (NO) release in dilated spermatic veins appears to negatively impact testicular function through oxidative stress pathways (17, 18). Interestingly, despite the established role of trace elements (Cu, Fe, Zn) in redox homeostasis, their concentrations remain stable pre- and post-varicocelectomy (19). Of particular clinical relevance is the observed postoperative elevation of asymmetric dimethylarginine (ADMA), an endogenous NOS inhibitor, which may represent a compensatory mechanism against NO-mediated oxidative damage (19). However, the field remains limited by the absence of comprehensive metabolomic investigations that could provide novel insights into the systemic metabolic consequences of varicocele.

In this study, we utilized blood samples from the local varicose veins as the primary subjects for metabolomic analysis, aiming to investigate the local metabolic characteristics of varicocele patients. By differentiating varicocele patients with normal fertility from those with male infertility based on seminal analysis parameters and clinical features, we compared the metabolic differences between the two groups. Our findings offer new insights into the pathological mechanisms underlying male infertility caused by VC and pave the way for novel strategies in the precise treatment of VC.

## Methods

### Patients and study groups

This study was a retrospective analysis conducted with the approval of the Hospital Medical Ethics Committee (Approval No. 2021-SR-574). All participants provided informed consent and signed consent forms. The study included patients diagnosed with VC who sought treatment at the Department of Urology, First Affiliated Hospital of Nanjing Medical University, between January 2021 and January 2023, as well as healthy volunteers who underwent health assessments during the same period. VC patients were categorized into the varicocele group (VC group) and further stratified into the varicocele fertile group (VF group) and varicocele infertile group (VI group) based on whether they had accompanying male infertility. Healthy volunteers were enlisted in the negative control group (NC group).

The inclusion criteria for the VC group were as follows: confirmation of varicocele diagnosis through clinical physical examination and ultrasound assessment, with indications for surgical intervention. In addition to the general inclusion criteria, participants in the VF group were required to meet the following criteria: adult patients who had fathered at least one child in the past 12 months, without any history of male infertility or infertility treatment. And participants in the VI group were required to meet the following criteria: adult patients meeting the diagnostic criteria for male infertility based on the guidelines of the American Society for Reproductive Medicine Practice Committee (20), with no history of other male infertility causes or infertility treatment or known female infertility factors in the partner. The inclusion criteria for healthy volunteers were as follows: confirmation of the absence of varicocele through clinical physical examination and ultrasound assessment, with no history of varicocele; normal semen analysis results; having fathered at least one child in the past 12 months, with no history of male infertility or infertility treatment.

Participants were excluded from this study if they presented with any of the following conditions: urinary or reproductive system infections, urinary system diseases diagnosed through urological examination, male genital diseases diagnosed through andrological examination, genetic defects, history of cryptorchidism, history of radiation therapy, history of chemotherapy, history of scrotal or inguinal trauma or surgery, current or recent (within 12 months) use of testosterone or similar synthetic metabolic steroids, current or recent use of medication such as antioxidants, moderate to severe liver or kidney dysfunction, or concomitant severe illnesses such as heart or lung failure, or systemic autoimmune diseases such as systemic lupus erythematosus. Additionally, participants who were lost to follow-up or unable to comply with follow-up were also excluded from the study.

All participants received thorough physical examinations administered by a highly experienced urological surgeon in a bright and comfortable examination room. Comprehensive semen analyses and scrotal Doppler ultrasound assessments were conducted for each participant. Semen analysis adhered to the guidelines outlined by the World Health Organization, while scrotal Doppler ultrasound, utilizing a linear high-frequency probe, was employed to evaluate varicocele diameter and blood reflux.

### Sample preparation for analysis

100 μL liquid sample was added to a 1.5 mL centrifuge tube with 400 μL solution (acetonitrile: methanol = 1:1(v:v)) to extract metabolites. The samples were mixed by vortex for 30 s and low-temperature sonicated for 30 min (5°C, 40 KHz). The samples were placed at -20°C for 30 min to precipitate the proteins. Then the samples were centrifuged for 15 min (4°C, 13000 g). The supernatant was removed and blown dry under nitrogen. The sample was then re-solubilized with 100 μL solution (acetonitrile: water = 1:1) and extracted by low-temperature ultrasonication for 5 min (5°C, 40 KHz), followed by centrifugation at 13000 g and 4°C for 10 min. The supernatant was transferred to sample vials for liquid chromatography-mass spectrometry (LC-MS) analysis.

As a part of the system conditioning and quality control process, a pooled quality control sample (QC) was prepared by mixing equal volumes of all samples. The QC samples were disposed and tested in the same manner as the analytic samples. It helped to represent the whole sample set, which would be injected at regular intervals (every 5–15 samples) in order to monitor the stability of the analysis.

The LC-MS/MS analysis of sample was conducted on a Thermo UHPLC-Q Exactive HF-X system equipped with an ACQUITY HSS T3 column (100 mm × 2.1 mm i.d., 1.8 μm; Waters, USA) at Majorbio Bio-Pharm Technology Co. Ltd. (Shanghai, China). The mobile phases consisted of 0.1% formic acid in water:acetonitrile (95:5, v/v) (solvent A) and 0.1% formic acid in acetonitrile:isopropanol:water (47.5:47.5, v/v) (solvent B). The flow rate was 0.40 mL/min and the column temperature was 40°C. The mass spectrometric data were collected using a Thermo UHPLC-Q Exactive HF-X Mass Spectrometer equipped with an electrospray ionization (ESI) source operating in positive mode and negative mode. The optimal conditions were set as followed: source temperature at 425°C; sheath gas flow rate at 50 arb; Aux gas flow rate at 13 arb; ion-spray voltage floating (ISVF) at -3500V in negative mode and 3500V in positive mode, respectively; Normalized collision energy, 20-40-60V rolling for MS/MS. Full MS resolution was 60000, and MS/MS resolution was 7500. Data acquisition was performed with the Data Dependent Acquisition (DDA) mode. The detection was carried out over a mass range of 70–1050 m/z.

### Untargeted metabolomics analysis

For participants in the NC group, peripheral venous blood samples were collected between 8:00 a.m. and 10:00 a.m. after an overnight fast of 8 hours. For participants in the VC group, the varicocele was surgically treated with open modified microsurgical varicocelectomy performed by the same surgeon (CQ). During the procedure, sterile syringes were used to collect blood from the severed end of the varicose veins and preserved in collection tubes.

The collected blood samples were allowed to clot by incubating them at 37°C for 1 hour in centrifuge tubes. Subsequently, they were centrifuged for 10 minutes (3000 rpm). The supernatant was transferred to clean centrifuge tubes and centrifuged for 10 minutes (4°C, 12000 rpm). The resulting supernatant was aliquoted into 1.5 mL centrifuge tubes, with each tube containing 0.2 mL of the supernatant. These aliquots were stored at -80°C and transported to Majorbio Bio-Pharm Technology Co. Ltd. (Shanghai, China) for subsequent sequencing and analysis.

Sample preparation for analysis and subsequent data processing can be referenced in **Supplementary File 1**. The data matrix obtained by searching database was uploaded to the Majorbio cloud platform (https://cloud.majorbio.com) for data analysis (21).

### Data analysis

The pretreatment of LC/MS raw data was performed by Progenesis QI (Waters Corporation, Milford, USA) software, and a three-dimensional data matrix in CSV format was exported. Internal standard peaks, as well as any known false positive peaks, were removed from the data matrix, deredundant and peak pooled. At the same time, the metabolites were identified by searching database, and the main databases were the HMDB (http://www.hmdb.ca/), Metlin (https://metlin.scripps.edu/) and Majorbio Database.

The raw data matrix was pre-processed on the Majorbio cloud platform (https://cloud.majorbio.com), which included filtration, sum normalization, and log10 transformation. Quality control was enforced by excluding metabolic features with a relative standard deviation (RSD) > 30% in QC samples.

Then, the R package “ropls” was used to perform principal component analysis (PCA), least partial squares discriminant analysis (PLS-DA), orthogonal least partial squares discriminant analysis (OPLS-DA), and permutation tests evaluating the stability of the model. The metabolites with VIP>1, P-value<0.05 were determined as significantly different metabolites based on the variable importance in the projection (VIP) obtained by the PLS-DA or OPLS-DA model and the P-value generated by Welch’s t test.

Differential metabolites were subsequently mapped to KEGG pathways for metabolic pathway enrichment analysis (http://www.genome.jp/kegg/). Enrichment analysis was conducted using the “scipy.stats” package (https://docs.scipy.org/doc/scipy/) in Python to identify the most relevant biological pathways associated with the experimental treatments.

### Statistical analysis

Statistical analyses were performed using SPSS 26.0 (SPSS Inc., Chicago, IL, USA). The Shapiro-Wilk test assessed data normality. Continuous variables were compared using Student’s t-test (normal distribution) or Mann-Whitney U test (non-normal distribution), while categorical variables used chi-square or Fisher’s exact tests. Paired comparisons employed paired t-test (normal) or Wilcoxon signed-rank test (non-normal). To control the increased risk of Type I errors due to multiple hypothesis testing, the False Discovery Rate (FDR) correction was applied using the Benjamini-Hochberg procedure. Statistical significance was set at *P*<0.05. All reported *p*-values for multiple comparisons are FDR-adjusted unless otherwise specified.

## Results

### Clinical demographics of the research cohort

From January 2021 to January 2023, a total of 40 patients with VC who underwent surgical treatment, as well as 35 healthy volunteers were enrolled in this study. Among the VC patients, 22 were assigned to the VF group, while 18 were assigned to the VI group. In accordance with the latest consensus among experts in the diagnosis and management of man infertility (22), among the 18 patients in the VI group, 12 were diagnosed with asthenozoospermia, 5 with oligoasthenozoospermia, and 1 with azoospermia. Clinical data of participants and relevant parameters from ultrasound and semen analysis are presented in **Table 1**. Patients in the VI group exhibited a significant increase in the diameter of the left spermatic vein, along with a marked decrease in total sperm count, sperm concentration, sperm progressive motility, and overall motility.

**Table 1:** Clinical and laboratorial characteristics of participants in this study.

Variables	C (*n* = 35)	VF (*n* = 22)	VI (*n* = 18)	*P*-value
Clinical characteristics				
Age (years), mean ± SD	23.97±6.73^a^	20.45±2.32^b^	29.89±7.33^c^	< 0.05^*^
Left varicocele grade %				
1	NA	22.7%	11.11%	0.37^†^
2		50.0%	38.9%	
3		23.7%	50.0%	
Right varicocele grade %				
1	NA	33.3%	28.6%	0.76^†^
2		66.7%	42.9%	
3		0%	28.6%	
Bilateral varicoceles %	NA	22.7%	38.9%	0.32^†^
Semen analysis parameters				
Total sperm count (10^6^/mL), median ± IQR	776.81±512.74^a^	769.62±713.01^a^	70.68±186.68^b^	< 0.001^§^
Sperm concentration (10^6^/mL), median ± IQR	179.35±119.61^a^	199.98±151.87^a^	25.31±64.42^b^	< 0.001^§^
Progressive motility %, mean ±SD	46.24±6.61^a^	46.42±6.54^a^	9.97±6.72^b^	< 0.001^*^
Total motility %, mean ± SD	81.39±8.25^a^	72.75±10.84^b^	19.39±11.82^c^	< 0.001^*^
Ultrasonography parameters				
Left varicose spermatic vein size by color Doppler (mm), median ± IQR	NA	2.84±0.36^a^	3.27±0.60^b^	< 0.05^*^
Right varicose spermatic vein size by color Doppler (mm), median ± IQR		2.50±0.42	2.99±0.69	0.16^*^

*SD* standard deviation, *IQR* interquartile range.

^*^Analysis of variance; ^†^Fischer’s exact test; ^§^Kruskal-Wallis test. ^a–c^ means in a row without common superscript letters differ (*P* < 0.05).

### Preprocessing and quality control of metabolomic data

**Figure 1:**
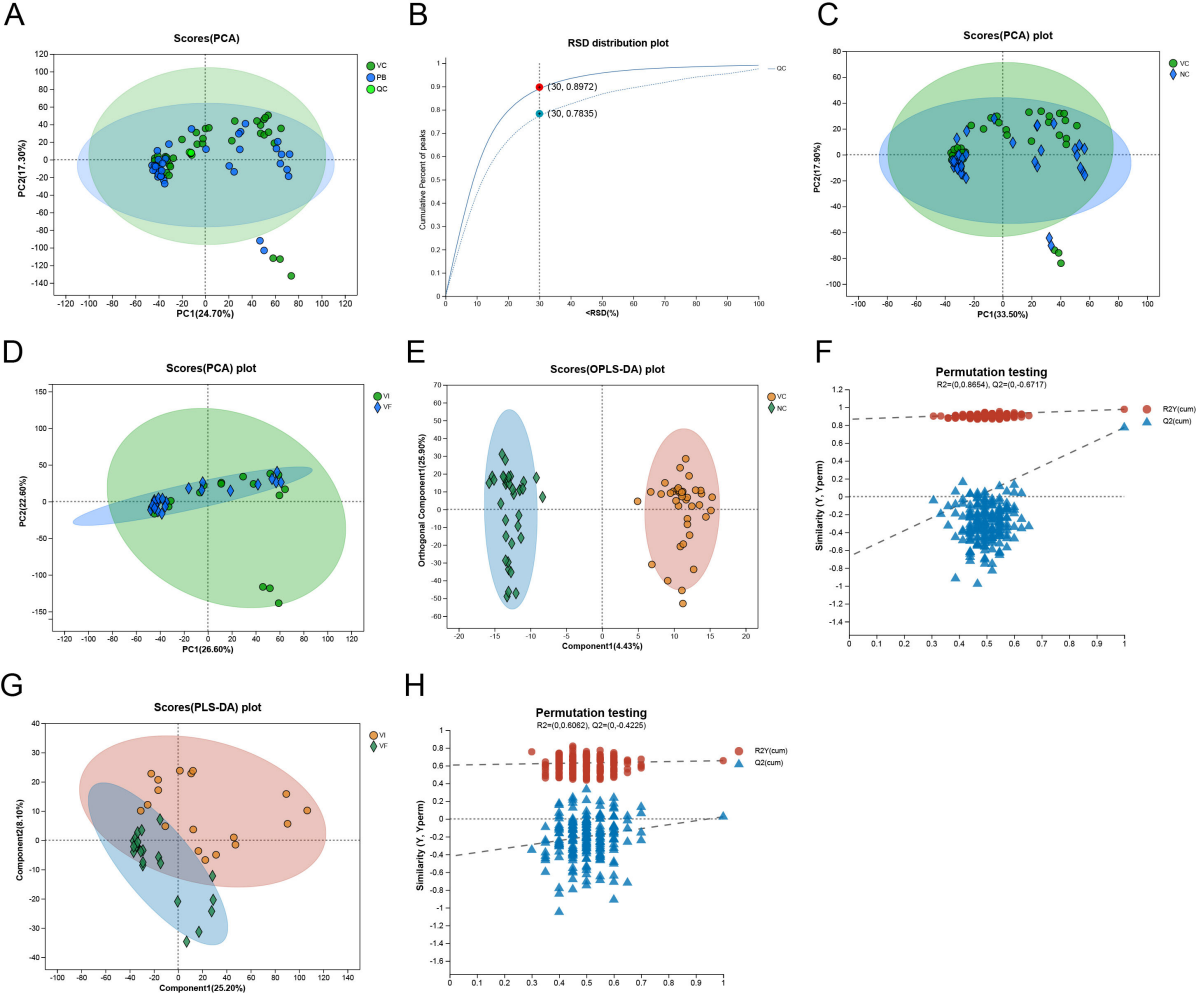
Evaluation of Experimental Data Quality and Establishment of Intergroup Difference Models. **(A)** PCA Analysis of the VC, NC, and QC Groups; **(B)** RSD Distribution of the QC Group; **(C)** PCA Analysis of the VC and NC Groups; **(D)** PCA Analysis of the VI and VF Groups; **(E)** OPLS-DA Analysis of the VC and NC Groups; **(F)** Permutation Test of OPLS-DA Model for the VC and NC Groups; **(G)** PLS-DA Analysis of the VI and VF Groups; **(H)** Permutation Test of PLS-DA Model for the VI and VF Groups.

After chromatographic separation of metabolites in serum, characteristic ion peak areas were measured in mass spectrometry. Different colored chromatographic peaks represent different metabolites. The matrix file identified through laboratory searches revealed 935 compounds in positive ion mode and 321 compounds in negative ion mode. To reduce errors introduced during experimentation and analysis, the raw data underwent preprocessing steps such as filtering, imputation, normalization, and logarithmic transformation. Consequently, the number of identified compounds decreased to 568 in positive ion mode and 229 in negative ion mode. The samples were categorized into three groups based on their sources: VC samples, NC samples, and quality control (QC) samples. The principal component analysis (PCA) plot in the positive and negative ion mixed mode showed that QC samples formed tight clusters (**Figure 1A**), indicating good experimental repeatability and reliability. After our preprocessing, the proportion of substances with a relative standard deviation (RSD) <30% among QC samples increased from 78.3% to 89.7%, demonstrating the stability and robustness of the experimental data (**Figure 1B**).

### Establishment of the metabolomic analysis models

To facilitate subsequent differential analysis, we first employed PCA to reduce the dimensionality of the metabolite abundance data. The results indicated that the PCA model could not distinguish between samples from the VC group and the NC group, nor between the VF group and the VI group (**Figures 1C, D**). Due to the significantly smaller sample size compared to the number of variables, we proceeded to construct and validate least partial squares discriminant analysis (PLS-DA) and orthogonal least partial squares discriminant analysis (OPLS-DA) models. The **Figure 1E** suggested that the samples from the NC group and the VC group were distinctly separated in the OPLS-DA model. We conducted 200 permutation tests on this model, which revealed an R2Y intercept of 0.865 and a Q2 intercept of -0.672 on the Y-axis (**Figure 1F**). The positive slope of the regression lines for Q2 and R2Y indicated that the model was stable and not overfitted. The **Figure 1G** illustrated that the samples from the VF group and the VI group were relatively separated in the PLS-DA model. The permutation test results for this model showed an R2Y intercept of 0.606 and a Q2 intercept of -0.423 on the Y-axis (**Figure 1H**). The positive slope of the regression lines for Q2 and R2Y indicated that the model was stable and not overfitted.

### Screening of differential metabolites and metabolic pathways

**Figure 2:**
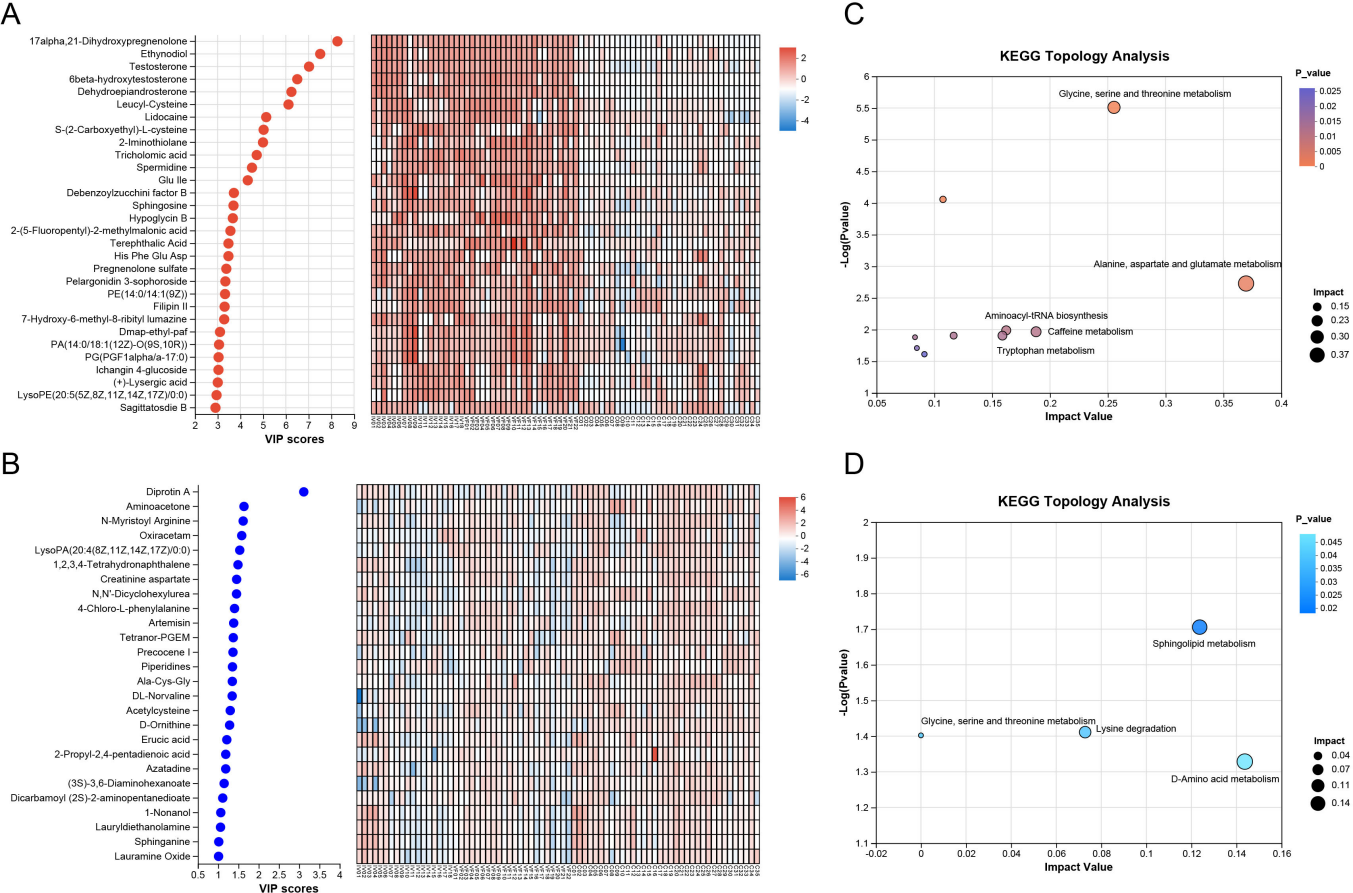
Differential Metabolite Screening and Enrichment and Topology Analyses of Pathways Between the VC and NC Groups. **(A, B)** Significantly **(A)** Upregulated and **(B)** Downregulated Metabolites in the VC Group (partial, ordered by descending VIP score); **(C, D)** Enriched Pathways of Significantly **(C)** Upregulated and **(D)** Downregulated Metabolites in the VC Group.

Using the aforementioned multivariate statistical methods, we successfully established analytical models that distinguish between the NC group and the VC group, as well as between the VF group and the VI group, indicating that significant metabolic differences exist between the groups, which may be associated with varicocele status and fertility. Under the screening criteria of P-value< 0.05, variable importance in the projection (VIP) value> 1, and fold change> 1, we identified a total of 195 metabolites with significantly upregulated expression and 26 metabolites with significantly downregulated expression in the VC group (7.5:1 ratio), indicating predominant pathway activation. VIP values reflect the importance of each variable in explaining the dependent variable and can be used to identify variables that contribute significantly to the model. In this study, we ranked the upregulated and downregulated metabolites based on their VIP values. According to **Figures 2A, B**, the top 10 compounds with the most significant contributions to distinguishing between the groups in the VC group, in terms of upregulated metabolites, were: 17alpha,21-Dihydroxypregnenolone, Ethynodiol, Testosterone, 6beta-hydroxytestosterone, Dehydroepiandrosterone, Leucyl-Cysteine, Lidocaine, S-(2-Carboxyethyl)-L-cysteine, 2-Iminothiolane, and Tricholomic acid. Exploring biological pathways may help elucidate the metabolic changes in varicocele. Therefore, we performed pathway enrichment and topological analysis of the upregulated and downregulated metabolites in the VC group based on KEGG. For the upregulated metabolites, we identified the top 5 significantly affected metabolic pathways, based on Impact value in descending order and P-value<0.05, as: alanine, aspartate and glutamate metabolism; glycine, serine and threonine metabolism; caffeine metabolism; aminoacyl-tRNA biosynthesis; and tryptophan metabolism (**Figure 2C**). For the downregulated metabolites, we enriched a total of 3 significantly affected metabolic pathways: D-amino acid metabolism; sphingolipid metabolism; and lysine degradation (**Figure 2D**).

**Figure 3:**
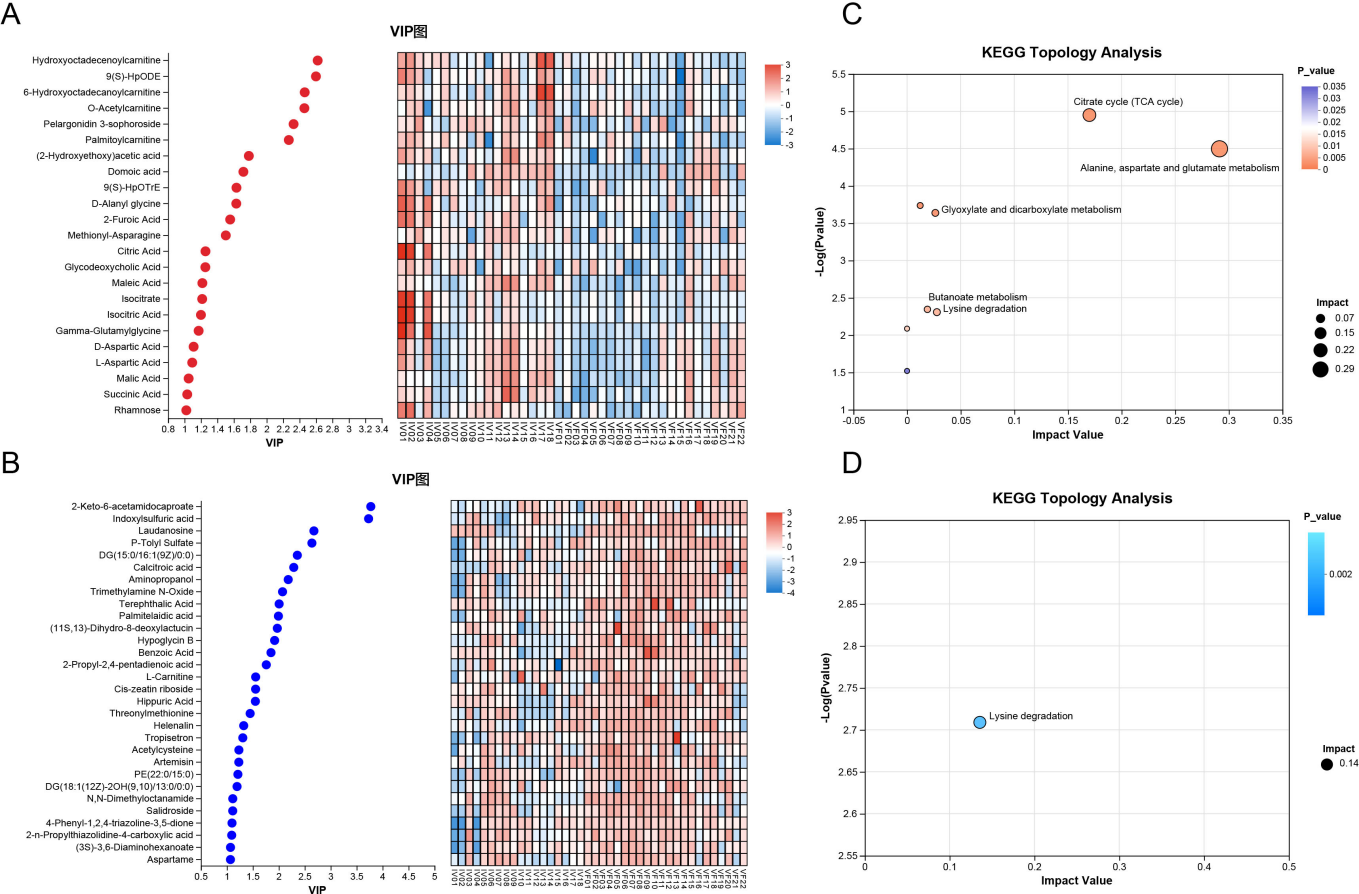
Differential Metabolite Screening and Enrichment and Topology Analyses of Pathways Between the VI and VF Groups. **(A, B)** Significantly **(A)** Upregulated and **(B)** Downregulated Metabolites in the VI Group (partial, ordered by descending VIP score); **(C, D)** Enriched Pathways of Significantly **(C)** Upregulated and **(D)** Downregulated Metabolites in the VI Group.

Subsequently, we conducted a differential expression analysis of the identified metabolites between the VI and VF groups. Applying the same screening criteria as previously described, we identified 23 metabolites that were significantly upregulated and 33 metabolites that were significantly downregulated in the VI group. The detailed results were shown in **Table 2**. Among the upregulated metabolites in the VI group, the top 10 metabolites that contributed most to distinguishing the VI group from the VF group were: Hydroxyoctadecenoylcarnitine, 9(S)-HpODE, 6-Hydroxyoctadecanoylcarnitine, O-Acetylcarnitine, Pelargonidin 3-sophoroside, Palmitoylcarnitine, (2-Hydroxyethoxy) acetic acid, Domoic acid, 9(S)-HpOTrE, and D-Alanyl glycine (**Figure 3A**). For the downregulated metabolites in the VI group, the key metabolites that most significantly differentiated the two groups were: 2-Keto-6-acetamidocaproate, Indoxylsulfuric acid, Laudanosine, P-Tolyl Sulfate, DG(15:0/16:1(9Z)/0:0), and Calcitroic acid (**Figure 3B**). Similarly, to explore the metabolic characteristics of varicocele-induced male infertility, we performed pathway analysis on differentially expressed metabolites in the VI group (P-value<0.05) using KEGG, identifying the most significantly affected pathways. The upregulated metabolites in the VI group were primarily enriched in the following metabolic pathways: Alanine, aspartate, and glutamate metabolism; Citrate cycle; Pyruvate metabolism; Glyoxylate and dicarboxylate metabolism; and Sulfur metabolism (**Figure 3C**). Conversely, the downregulated metabolites in the VI group were mainly enriched in the lysine degradation pathway (**Figure 3D**).

**Table 2:** Differentially expressed and most important metabolites between VI and VF groups and their expression trends in the VI Group.

Metabolite	VIP	*P-value*	Regulate
Hydroxyoctadecenoylcarnitine	2.621	0.006	up
9(S)-HpODE	2.600	0.002	up
6-Hydroxyoctadecanoylcarnitine	2.463	0.008	up
O-Acetylcarnitine	2.459	0.034	up
Pelargonidin 3-sophoroside	2.328	0.004	up
Palmitoylcarnitine	2.270	0.026	up
(2-Hydroxyethoxy)acetic acid	1.783	0.003	up
Domoic acid	1.717	0.019	up
9(S)-HpOTrE	1.633	0.025	up
D-Alanyl glycine	1.630	0.007	up
2-Furoic Acid	1.556	0.003	up
Methionyl-Asparagine	1.503	0.029	up
Citric Acid	1.256	0.013	up
Glycodeoxycholic Acid	1.254	0.048	up
Maleic Acid	1.218	0.011	up
Isocitrate	1.216	0.012	up
Isocitric Acid	1.200	0.015	up
Gamma-Glutamylglycine	1.172	0.018	up
D-Aspartic Acid	1.111	0.012	up
L-Aspartic Acid	1.094	0.015	up
Malic Acid	1.051	0.019	up
Succinic Acid	1.034	0.018	up
Rhamnose	1.022	0.029	up
2-Keto-6-acetamidocaproate	3.773	0.001	down
Indoxylsulfuric acid	3.731	0.003	down
Laudanosine	2.678	0.010	down
P-Tolyl Sulfate	2.639	0.011	down
DG(15:0/16:1(9Z)/0:0)	2.358	0.002	down
Calcitroic acid	2.289	0.001	down
Aminopropanol	2.183	0.007	down
Trimethylamine N-Oxide	2.073	0.005	down
Terephthalic Acid	2.008	0.005	down
Palmitelaidic acid	1.994	0.006	down
(11S,13)-Dihydro-8-deoxylactucin	1.972	0.014	down
Hypoglycin B	1.921	0.001	down
Benzoic Acid	1.851	0.011	down
2-Propyl-2,4-pentadienoic acid	1.760	0.021	down
L-Carnitine	1.555	0.050	down
Cis-zeatin riboside	1.554	0.045	down
Hippuric Acid	1.550	0.024	down
Threonylmethionine	1.449	0.004	down
Helenalin	1.325	0.024	down
Tropisetron	1.308	0.012	down
Acetylcysteine	1.232	0.017	down
Artemisin	1.231	0.043	down
PE(22:0/15:0)	1.213	0.025	down
DG(18:1(12Z)-2OH(9,10)/13:0/0:0)	1.197	0.048	down
N,N-Dimethyloctanamide	1.114	0.014	down
Salidroside	1.113	0.013	down
4-Phenyl-1,2,4-triazoline-3,5-dione	1.100	0.031	down
2-n-Propylthiazolidine-4-carboxylic acid	1.094	0.047	down
(3S)-3,6-Diaminohexanoate	1.071	0.044	down
Aspartame	1.070	0.018	down
5-Hydroxyindoleacetylglycine	1.045	0.018	down
5-Aminopentanoic acid	1.010	0.048	down
Asp Leu	1.009	0.021	down

To clarify the metabolic differences associated with fertility status, we further compared the quantitative levels of oxidative-stress-related metabolites among the VI, VF, and NC groups. NAC levels were significantly reduced in varicocele patients, with the VI group showing the lowest abundance (5.58 ± 0.20), whereas the VF and NC groups showed similar but higher levels (5.84 ± 0.18 and 5.80 ± 0.22, respectively). In contrast, acylcarnitine metabolism displayed a fertility-dependent increasing trend. Propionylcarnitine exhibited mean values of 4.66 ± 0.58, 4.92 ± 0.34, and 4.96 ± 0.25 in NC, VF, and VI groups, respectively. A similar pattern was observed in other oxidative-stress-associated metabolites: succinic acid increased stepwise from NC (4.46 ± 0.14) to VF (4.69 ± 0.18) and VI (4.79 ± 0.16), and aspartic acid increased from 3.92 ± 0.43 (NC) to 4.32 ± 0.38 (VF) and 4.63 ± 0.40 (VI). Collectively, these findings suggest a consistent gradient in oxidative-stress–related metabolic disturbances that correlates with the severity of fertility impairment.

The Venn diagram analysis (**Supplementary Figure S1**) uncovered a distinctive metabolic transition pattern during varicocele progression from subclinical status (VC) to infertility (VI). Among 221 differentially expressed metabolites in VC patients (195 upregulated, 26 downregulated), only 15 metabolites (11 up- and 4 down-regulated) maintained abnormalities in VI stage, demonstrating a remarkable metabolic filtering effect.

### Confirmation of the mechanisms underlying varicocele-induced male infertility

There are currently numerous theories attempting to explain the mechanisms by which varicocele causes male infertility. Based on our metabolomic results, we sought to evaluate the consistency of our findings with the renal and adrenal metabolite reflux theory as well as the oxidative stress theory.

**Figure 4:**
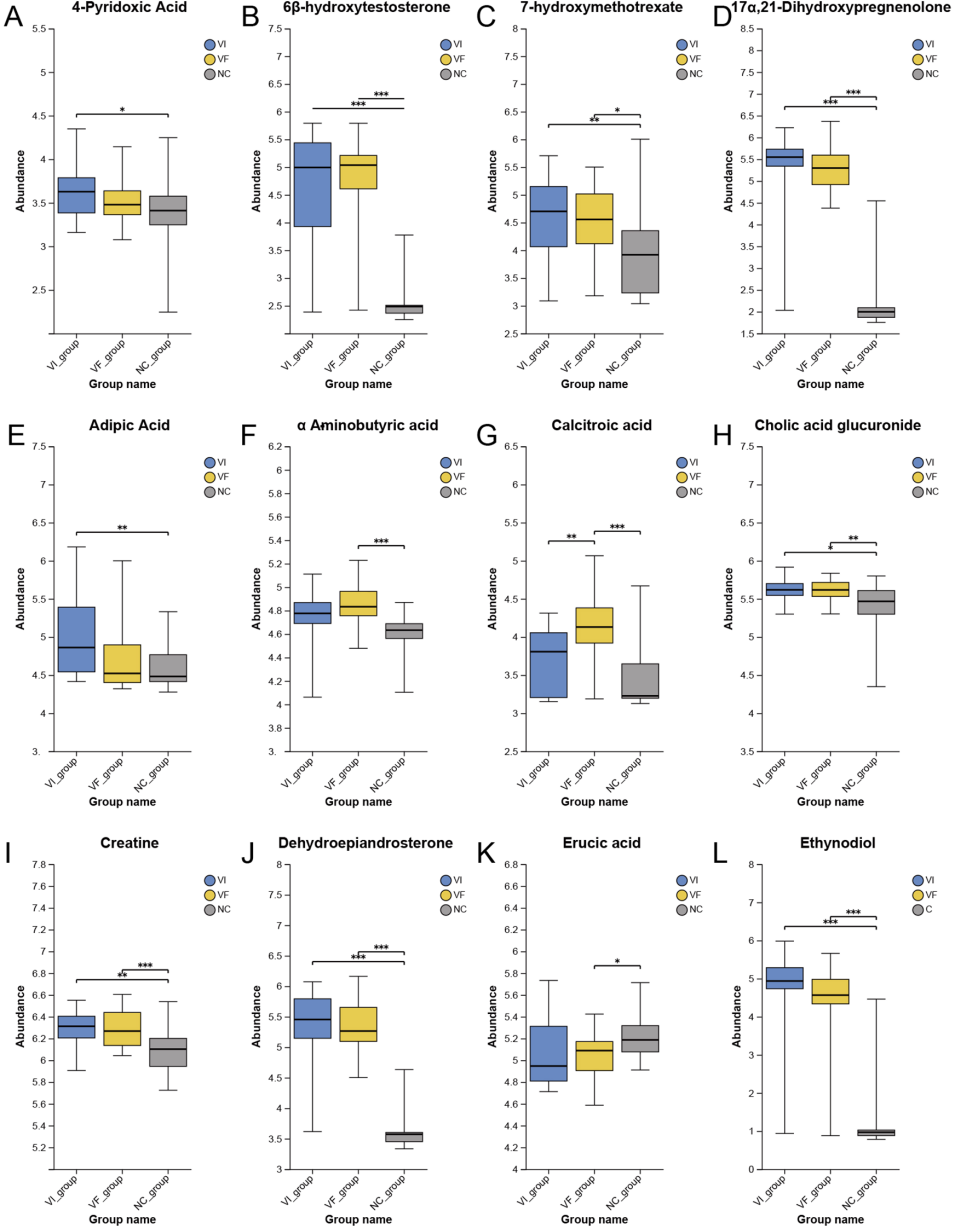
Differential Expression of Metabolites Derived from Kidney and Adrenal Glands Among the VI, VF, and NC Groups, Part I. **(A)** 4-Pyridoxic Acid; **(B)** 6β-hydroxytestosterone; **(C)** 7-hydroxymethotrexate; **(D)** 17α,21-Dihydroxypregnenolone; **(E)** Adipic Acid; **(F)** α-Aminobutyric acid; **(G)** Calcitroic acid; **(H)** Cholic acid glucuronide; **(I)** Creatine; **(J)** Dehydroepiandrosterone; **(K)** Erucic acid; **(L)** Ethynodiol.

**Figure 5:**
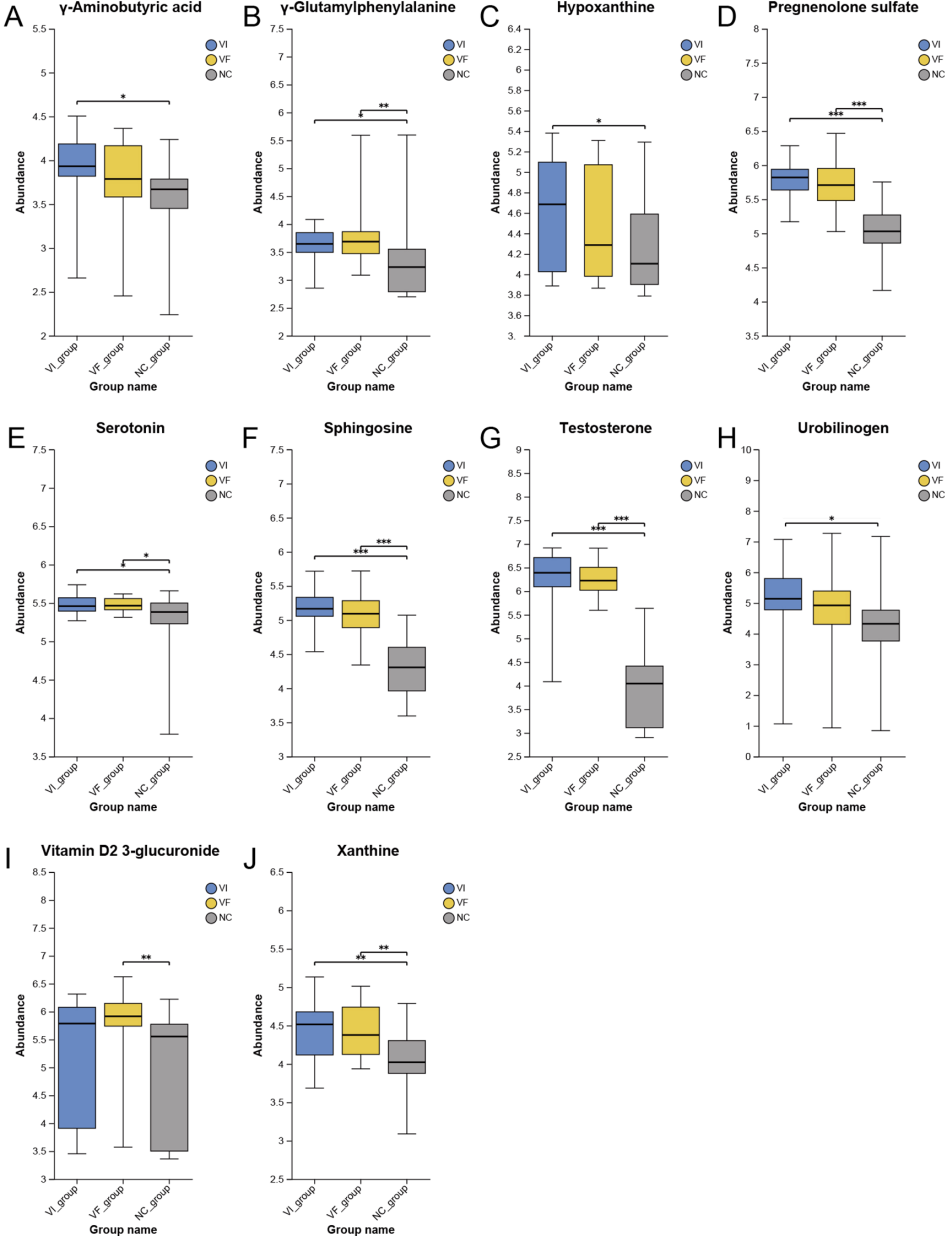
Differential Expression of Metabolites Derived from Kidney and Adrenal Glands Among the VI, VF, and NC Groups, Part II. **(A)** γ-Aminobutyric acid; **(B)** γ-Glutamylphenylalanine; **(C)** Hypoxanthine; **(D)** Pregnenolone sulfate; **(E)** Serotonin; **(F)** Sphingosine; **(G)** Testosterone; **(H)** Urobilinogen; **(I)** Vitamin D 2 3-glucuronide; **(J)** Xanthine.

The Human Metabolome Database (HMDB, https://hmdb.ca/) is a free electronic database that contains detailed information about small molecule metabolites found in the human body. Based on the previously identified set of differentially expressed metabolites representing local metabolic characteristics of VC, we searched the HMDB for each metabolite to obtain their metabolic pathways and locations. Out of 221 metabolites, we identified 22 that might originate from the adrenal gland or kidneys (**Figures 4**, **5**). Among these, only calcitroic acid exhibited differential expression between the VI and VF groups, but its impact on male fertility has not been clearly reported.

**Figure 6:**
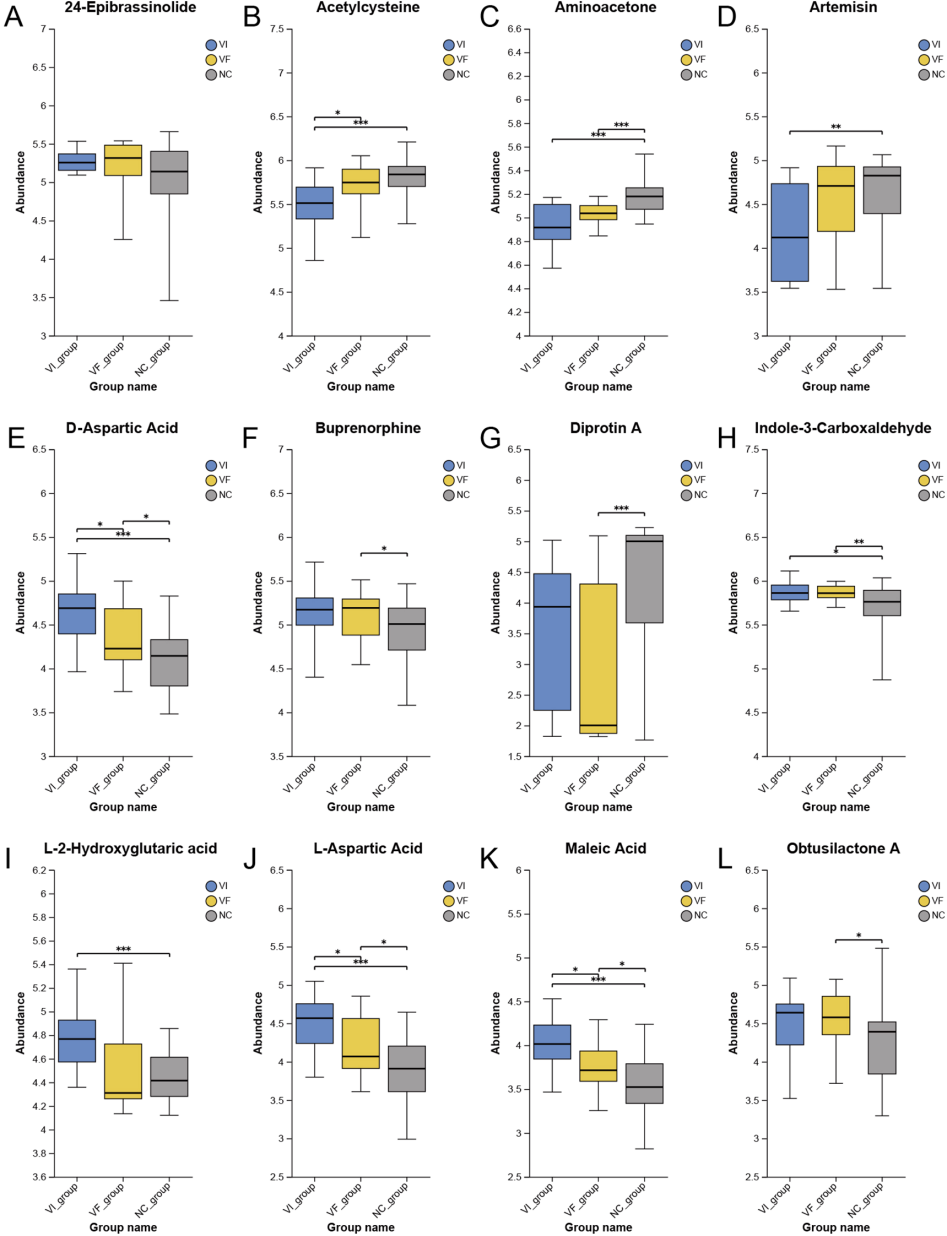
Differential Expression of Metabolites with Oxidative Stress and Inflammatory Functions Among the VI, VF, and NC Groups, Part I. **(A)** 24-Epibrassinolide; **(B)** Acetylcysteine; **(C)** Aminoacetone; **(D)** Artemisin; **(E)** D-Aspartic Acid; **(F)** Buprenorphine; **(G)** Diprotin A; **(H)** Indole-3-Carboxaldehyde; **(I)** L-2-Hydroxyglutaric acid; **(J)** L-Aspartic Acid; **(K)** Maleic Acid; **(L)** Obtusilactone A.

**Figure 7:**
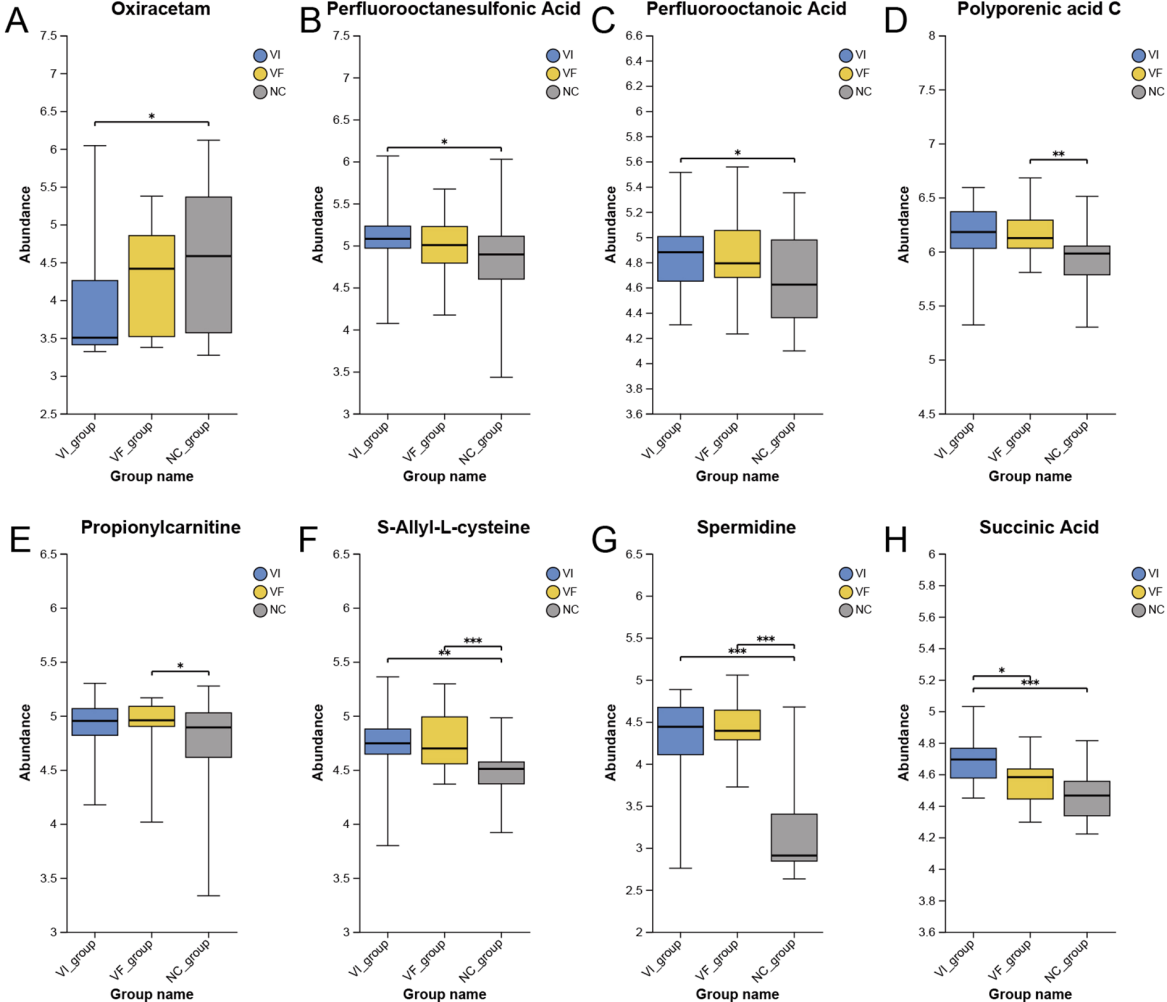
Differential Expression of Metabolites with Oxidative Stress and Inflammatory Functions Among the VI, VF, and NC Groups, Part II. **(A)** Oxiracetam; **(B)** Perfluorooctanesulfonic Acid; **(C)** Perfluorooctanoic Acid; **(D)** Polyporenic acid C; **(E)** Propionylcarnitine; **(F)** S-Allyl-L-cysteine; **(G)** Spermidine; **(H)** Succinic Acid.

PubMed (https://pubmed.ncbi.nlm.nih.gov) is a free biomedical abstract database that links to various full-text databases. To further explore the oxidative stress hypothesis, we similarly conducted a comprehensive literature search on each metabolite in the differentially expressed metabolite set. For each metabolite, we conducted a thorough literature search in the PubMed database using its synonyms obtained from the HMDB database, to investigate whether there have been relevant studies on oxidative stress, inflammation, or other related aspects. Out of the 221 metabolites, we identified a total of 20 metabolites that are associated with oxidative stress or inflammation in the human body or disease states (excluding the 22 metabolites related to renal and adrenal origins mentioned in the first part of this section). Detailed information on these metabolites could be found in **Table 3**. The expression profiles of corresponding metabolites in the VI, VF, and NC groups were depicted in **Figures 6** and **7**. Among the 20 metabolites related to oxidative stress and inflammation, significant differences (*P<*0.05) in the abundance of five metabolites—D-aspartic acid, L-aspartic acid, maleic acid, succinic acid, and acetylcysteine—were observed between the VI and VF groups. Maleic acid, being an exogenous substance, cannot be synthesized by the human body itself. Hence, exploring the mechanisms of action of the remaining four endogenous metabolites in oxidative stress may unveil novel therapeutic targets for male infertility attributed to varicocele.

**Table 3:** Detected xenobiotics in this research.

Compound Name	HMDB	Formula
Artemisin	HMDB0248621	C15H18O4
Aspartame	HMDB0001894	C14H18N2O5
Benzaldehyde	HMDB0006115	C7H6O
Benzene	HMDB0256717	C11H14O2
Benzoic Acid	HMDB0035268	C10H12O2
Chlorobenzene	HMDB0041855	C6H5Cl
Clarithromycin	HMDB0015342	C38H69NO13
Cryptophycin	HMDB0242628	C35H43ClN2O8
Cyclophosphamide	HMDB0014672	C7H15Cl2N2O2P
Dioctyl phthalate	HMDB0251427	C24H38O4
Domoic acid	HMDB0033939	C15H21NO6
Ephedrine	HMDB0015451	C10H15NO
Gymnodimine	HMDB0041430	C32H45NO4
Ibuprofen	HMDB0001925	C13H18O2
Ketoprofen	HMDB0015144	C16H14O3
Lauroylcarnitine	HMDB0002250	C19H37NO4
Lidocaine	HMDB0014426	C14H22N2O
Maleic Acid	HMDB0303644	C4H4O4
N,N'-Dicyclohexylurea	HMDB0244166	C13H24N2O
Octaethyleneglycol monododecyl ether	HMDB0246126	C28H58O9
Oxymetholone	HMDB0256016	C21H32O3
Perfluorooctanesulfonic Acid (PFOS)	–	C8HF17O3S
Perfluorooctanoic Acid (PFOA)	HMDB0256329	C8HF15O2
Piperine	HMDB0029377	C17H19NO3
Sorbitan palmitate	HMDB0029887	C22H42O6
Theophylline	HMDB0001889	C7H8N4O2
Thiram	HMDB0259039	C6H12N2S4
Toluene	HMDB0034168	C7H8
Triton X-100	HMDB0259285	C16H26O2
Vitamin D2 3-glucuronide	HMDB0010344	C34H52O7

## Discussion

Studies on VC reported an incidence rate of approximately 15% in the general population (23). Prior research has shown that VC can manifest in various detrimental effects on male reproductive health, including decreased sperm vitality, reduced count, structural anomalies, diminished testicular volume, and impaired Leydig cell function (24). However, there are still different arguments about the potential mechanism. This study represents the first metabolomic investigation using spermatic vein blood from varicocele (VC) patients, aiming to elucidate local metabolic alterations and their relevance to fertility (25–27). Compared with peripheral circulation and fertile individuals, we identified pronounced differences in metabolites involved in steroidogenesis, amino acid metabolism, and oxidative stress pathways. These findings suggest that varicocele creates a unique biochemical microenvironment potentially unfavorable to spermatogenesis.

In the comparison between the VC and NC groups, we identified 221 differentially expressed metabolites, predominantly belonging to steroids, amino acids, and organic acids. The marked predominance of upregulated metabolites may reflect compensatory metabolic adaptation to venous stasis and hypoxia. Steroid hormones and their precursors—including testosterone, 6β-hydroxytestosterone, and pregnenolone sulfate—were significantly upregulated in the VC group. This may reflect increased steroidogenic activity within the testis or adrenal-derived hormonal reflux due to venous insufficiency (28). Such alterations could contribute to endocrine dysregulation in VC patients and may have diagnostic implications. In addition, the upregulated metabolites in the VC group were primarily enriched in pathways related to amino acid metabolism, such as alanine, aspartate, and glutamate metabolism, as well as glycine, serine, and threonine metabolism. The metabolites enriched in the pathway of alanine, aspartate, and glutamate metabolism included gamma-aminobutyric acid (GABA), succinate, and aspartate. These metabolic alterations may lead to increased reactive oxygen species generation and oxidative stress, accompanied by a compensatory increase in anti-inflammatory effects, which may affect local spermatogenesis.

In addition, amino acid metabolic pathways—particularly alanine, aspartate, and glutamate metabolism—were enriched in the infertile subgroup. Within this pathway, the expression of aspartic acid (Asp) was relatively elevated in the VI cases. It is important to note that this observation indicates a correlation between elevated Asp levels and the infertile phenotype, which does not necessarily imply a direct causal relationship. Asp is an α-amino acid with two enantiomers—L-aspartic acid (L-Asp) and D-aspartic acid (D-Asp). Current research on the impact of Asp on male fertility primarily focuses on D-Asp. Asp, especially its D-enantiomer, plays complex roles in male reproduction by regulating the hypothalamic–pituitary–gonadal axis and promoting spermatogonial proliferation (29, 30). However, in the pathological context of varicocele, the observed elevation of D-Asp might be associated with adverse effects. One plausible hypothesis is that excessive levels of D-Asp could contribute to oxidative stress via hydrogen peroxide generation, potentially explaining the paradox of high D-Asp levels without improved testosterone production in infertile VC patients. The correlation between elevated D-Asp levels and infertility suggests its potential utility as a novel biomarker, which, if validated in plasma or seminal plasma, could aid in the clinical stratification of varicocele patients and the prediction of their reproductive prognosis. Through the action of D-Asp oxidase, D-Asp undergoes degradation into oxaloacetate, ammonia, and hydrogen peroxide, leading to oxidative stress (31). Studies reveal that elevated doses of D-Asp notably escalate levels of reactive oxygen species (ROS) and peroxides in the cytoplasm of rat testicular cells, alongside heightened activity of the antioxidant system, indicative of cellular oxidative stress (32) (33). The core concept of the oxidative stress theory in male infertility induced by VC posited that VC led to an increase in ROS or a reduction in overall antioxidant capacity within the male reproductive system. This imbalance resulted in lipid peroxidation, protein denaturation, and DNA damage, ultimately impairing spermatogenesis and causing sperm damage (34).

In the validation of the oxidative stress theory, we discovered differential expression of the oxidative stress-related metabolite between the VF and VI groups. Markers of oxidative stress and mitochondrial dysfunction were also evident. Decreased N-acetylcysteine levels and elevated long-chain acylcarnitines—such as palmitoylcarnitine—suggest impaired redox buffering and energy metabolism. In this study, NAC expression progressively decreased significantly from the NC group to the VF group and then to the VI group, indicating a reduction in local antioxidant capacity in the varicocele cohorts, particularly among those with male infertility. Among the differential metabolites between the VI and VF groups, we also noted an upregulation of acylcarnitines, which exhibited high VIP scores. Previous studies had shown that long-chain acylcarnitines, such as palmitoylcarnitine, could interfere with mitochondrial respiratory chain transfer in a dose-dependent manner, inducing mitochondrial membrane hyperpolarization and ROS production (35). Therefore, the observed upregulation of acylcarnitine expression in the VI group may reflect underlying metabolic characteristics associated with redox imbalance and energy metabolism disruption. Based on the metabolite profile and supported by previous literature, we infer that these changes are indicative of mitochondrial dysfunction, which lend support to the hypothesis that VC-induced infertility could be partially mediated by oxidative damage and mitochondrial dysfunction within the testes. These findings not only reinforce the role of oxidative stress in varicocele pathophysiology but also highlight these metabolites as potential targets for adjunct antioxidant therapy, which could be evaluated in future studies for improving semen parameters in patients opting for non-surgical management or as a neoadjuvant to varicocelectomy.

It is important to emphasize that oxidative stress is not an etiological mechanism initiating varicocele but rather a downstream pathological consequence triggered by venous stasis, hyperthermia, and hypoxia within the pampiniform plexus. Excessive ROS generation and impaired antioxidant buffering subsequently induce lipid peroxidation, mitochondrial injury, and germ-cell DNA fragmentation, ultimately contributing to impaired spermatogenesis. The concurrent elevation of steroid hormone intermediates and oxidative stress–related metabolites suggests that these processes are biologically interconnected rather than independent. ROS has been shown to disrupt mitochondrial cholesterol transport via StAR, impair CYP11A1, CYP17A1, and 3β-HSD activity, and alter LH receptor signaling, all of which compromise testosterone synthesis. Meanwhile, mitochondrial dysfunction—indicated by the accumulation of long-chain acylcarnitines—may further enhance ROS production, creating a vicious cycle that exacerbates testicular metabolic stress.

Admittedly, this study has certain limitations. The sample size was limited, which may increase the risk of sampling bias and reduce the statistical power to detect significant effects. Furthermore, potential confounders such as dietary habits and body mass index (BMI) were not accounted for in the analysis. Both diet and BMI are recognized as significant factors influencing metabolic profiles and reproductive outcomes (36, 37). Therefore, future studies with larger sample sizes should prospectively collect and adjust for these potential confounders to validate the conclusions of this research. Additionally, future experiments could also include peripheral blood samples and analyses from VC patients, potentially revealing more significant findings. While VF inclusion diverges from therapeutic guidelines, it enabled mechanistic insights. Future studies should validate findings in guideline-defined cohorts. Additionally, some key metabolites identified in this study need precise quantification through targeted metabolomics, as well as verification of their roles in the development and progression of VC via cell or animal experiments. While our analysis retained some xenobiotics reflecting clinical realities (**Supplementary File 2**), future studies should implement stricter medication screening. Future studies should integrate spermatic vein blood, seminal plasma, and testicular biopsies to map metabolite trafficking across the blood-testis barrier in VC. Lastly, while this study focused on semen analysis parameters to assess male infertility, future research could include parameters such as varicocele grade, testicular volume and diameter to provide a more comprehensive evaluation of the infertility status, facilitating more accurate phenotypic analysis. Another limitation of this study is the lack of paired peripheral blood sampling from varicocele patients. Our primary aim was to characterize local metabolic alterations within the spermatic vein rather than to examine intra-individual systemic–local differences. Because peripheral blood metabolites are strongly influenced by diet, lifestyle, and multi-organ metabolic processes, they may not accurately represent local testicular metabolic disturbances. Moreover, in healthy individuals, peripheral blood provides a valid surrogate for testicular venous metabolism, as collecting spermatic vein blood from healthy subjects is impractical. Future prospective studies incorporating paired sampling may provide additional mechanistic insights.

## Conclusion

In conclusion, this study found that VC patients exhibit increased synthesis and metabolism of steroid hormone-related metabolites, along with elevated amino acid metabolism linked to cell proliferation, energy metabolism, and oxidative stress. While D-Asp is known to promote male fertility, high levels may cause oxidative stress and cellular damage, negatively impacting the reproductive system. Although the renal and adrenal metabolite reflux hypothesis lacked sufficient evidence, the oxidative stress hypothesis was well-supported. Some oxidative stress-related metabolites may serve as therapeutic targets for VC-induced male infertility.

## Data Availability

The datasets presented in this study can be found in online repositories. The names of the repository/repositories and accession number(s) can be found in the article/**Supplementary Material**.

## Supplementary material

Supplementary Figure 1 Overlap analysis of differentially expressed metabolites across varicocele progression stages. A) Upregulated overlap between VC/NC (n=195) and VI/VF (n=23). B) Downregulated overlap between VC/NC (n=26) and VI/VF (n=33).
