# Supplementary figures and images for: Metabolomic profiling of varicocele-induced male infertility: insights from spermatic vein blood analysis
Source: Front Endocrinol (Lausanne). 2026 Jan 7;16:1682362. doi: 10.3389/fendo.2025.1682362 (PMC12819268; doi:10.3389/fendo.2025.1682362)

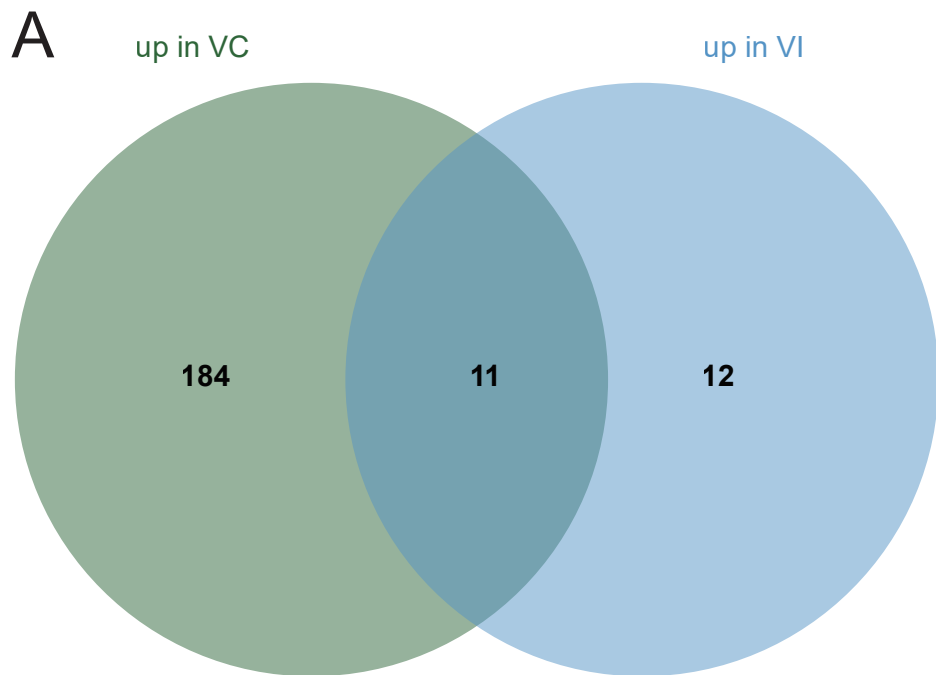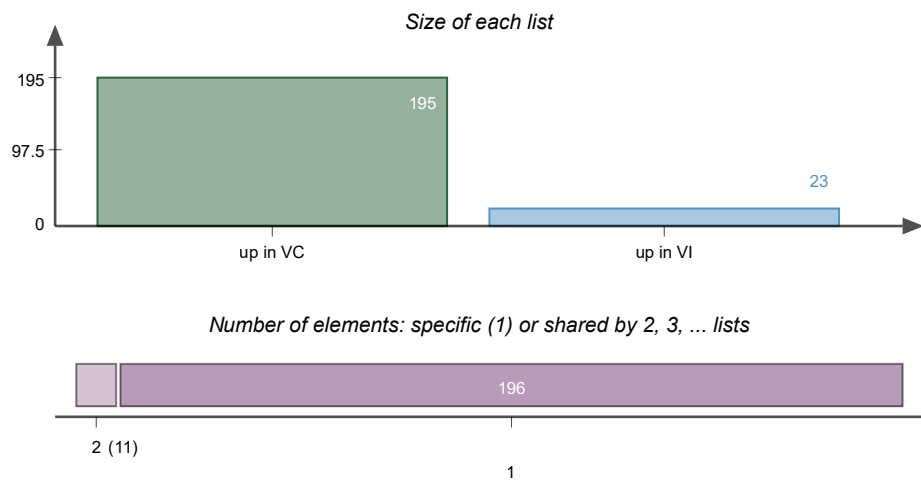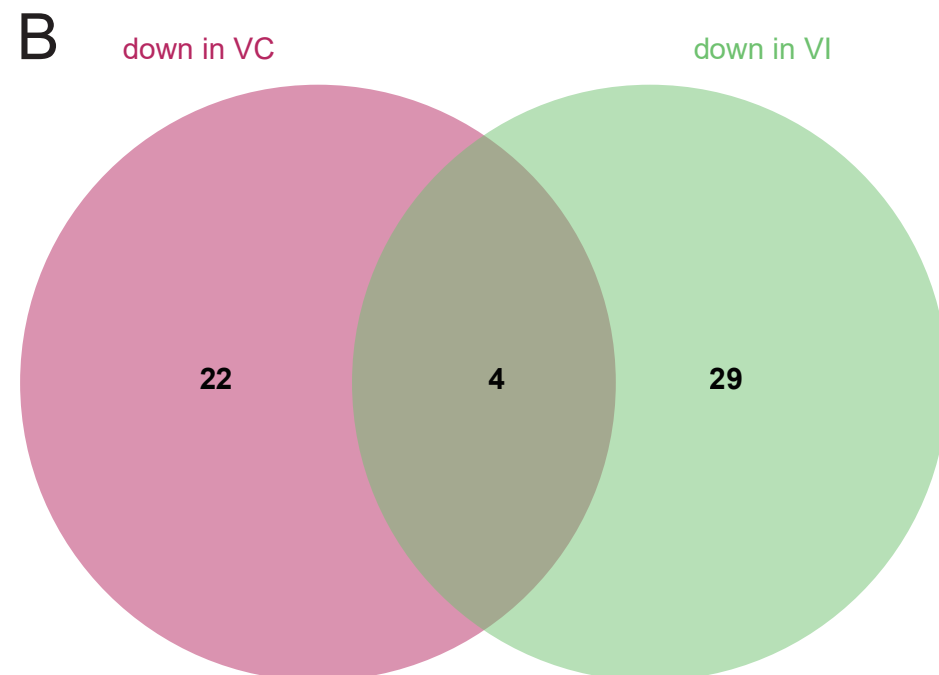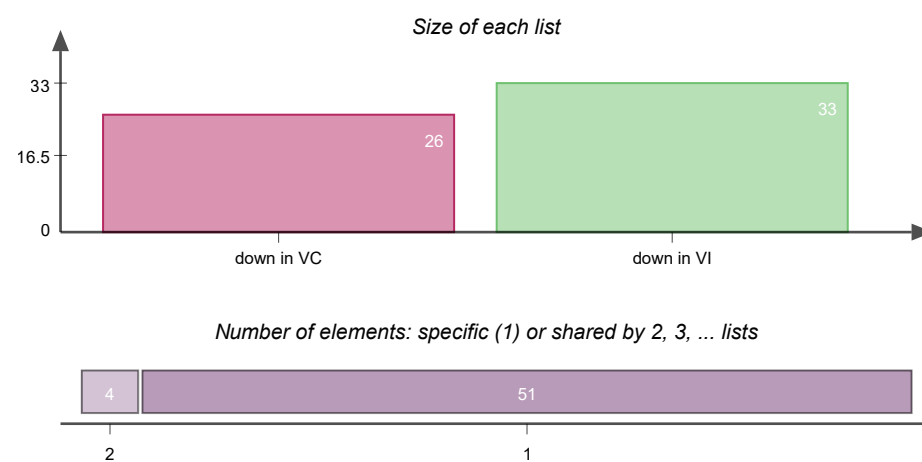

Supplement: Supplementary Figure 1 — Overlap analysis of differentially expressed metabolites across varicocele progression stages. A) Upregulated overlap between VC/NC (n=195) and VI/VF (n=23). B) Downregulated overlap between VC/NC (n=26) and VI/VF (n=33). [file Image1.pdf]
