# Supplementary material for: Metabolomic profiling of varicocele-induced male infertility: insights from spermatic vein blood analysis
Source: Front Endocrinol (Lausanne). 2026 Jan 7;16:1682362. doi: 10.3389/fendo.2025.1682362 (PMC12819268; doi:10.3389/fendo.2025.1682362)
Supplement: Supplementary file 2 [file DataSheet1.docx]

Supplementary Table 1. Detected xenobiotics in this research.

| **Compound Name** | **HMDB** | **Formula** |
| --- | --- | --- |
| Artemisin | HMDB0248621 | C15H18O4 |
| Aspartame | HMDB0001894 | C14H18N2O5 |
| Benzaldehyde | HMDB0006115 | C7H6O |
| Benzene | HMDB0256717 | C11H14O2 |
| Benzoic Acid | HMDB0035268 | C10H12O2 |
| Chlorobenzene | HMDB0041855 | C6H5Cl |
| Clarithromycin | HMDB0015342 | C38H69NO13 |
| Cryptophycin | HMDB0242628 | C35H43ClN2O8 |
| Cyclophosphamide | HMDB0014672 | C7H15Cl2N2O2P |
| Dioctyl phthalate | HMDB0251427 | C24H38O4 |
| Domoic acid | HMDB0033939 | C15H21NO6 |
| Ephedrine | HMDB0015451 | C10H15NO |
| Gymnodimine | HMDB0041430 | C32H45NO4 |
| Ibuprofen | HMDB0001925 | C13H18O2 |
| Ketoprofen | HMDB0015144 | C16H14O3 |
| Lauroylcarnitine | HMDB0002250 | C19H37NO4 |
| Lidocaine | HMDB0014426 | C14H22N2O |
| Maleic Acid | HMDB0303644 | C4H4O4 |
| N,N'-Dicyclohexylurea | HMDB0244166 | C13H24N2O |
| Octaethyleneglycol monododecyl ether | HMDB0246126 | C28H58O9 |
| Oxymetholone | HMDB0256016 | C21H32O3 |
| Perfluorooctanesulfonic Acid (PFOS) | - | C8HF17O3S |
| Perfluorooctanoic Acid (PFOA) | HMDB0256329 | C8HF15O2 |
| Piperine | HMDB0029377 | C17H19NO3 |
| Sorbitan palmitate | HMDB0029887 | C22H42O6 |
| Theophylline | HMDB0001889 | C7H8N4O2 |
| Thiram | HMDB0259039 | C6H12N2S4 |
| Toluene | HMDB0034168 | C7H8 |
| Triton X-100 | HMDB0259285 | C16H26O2 |
| Vitamin D2 3-glucuronide | HMDB0010344 | C34H52O7 |
