# Supplementary material for: Metabolomic profiling of varicocele-induced male infertility: insights from spermatic vein blood analysis
Source: Front Endocrinol (Lausanne). 2026 Jan 7;16:1682362. doi: 10.3389/fendo.2025.1682362 (PMC12819268; doi:10.3389/fendo.2025.1682362)
Supplement: Supplementary file 3 [file Supplementaryfile1.docx]

**Methods**

**Sample preparation for analysis**

100 μL liquid sample was added to a 1.5 mL centrifuge tube with 400 μL solution (acetonitrile: methanol = 1:1(v:v)) containing 0.02 mg/mL internal standard (L-2-chlorophenylalanine) to extract metabolites. The samples were mixed by vortex for 30 s and low-temperature sonicated for 30 min (5°C, 40 KHz). The samples were placed at -20°C for 30 min to precipitate the proteins. Then the samples were centrifuged for 15 min (4°C, 13000 g). The supernatant was removed and blown dry under nitrogen. The sample was then re-solubilized with 100 μL solution (acetonitrile: water = 1:1) and extracted by low-temperature ultrasonication for 5 min (5°C, 40 KHz), followed by centrifugation at 13000 g and 4°C for 10 min. The supernatant was transferred to sample vials for liquid chromatography-mass spectrometry (LC-MS) analysis.

As a part of the system conditioning and quality control process, a pooled quality control sample (QC) was prepared by mixing equal volumes of all samples. The QC samples were disposed and tested in the same manner as the analytic samples. It helped to represent the whole sample set, which would be injected at regular intervals (every 5-15 samples) in order to monitor the stability of the analysis.

The LC-MS/MS analysis of sample was conducted on a Thermo UHPLC-Q Exactive HF-X system equipped with an ACQUITY HSS T3 column (100 mm × 2.1 mm i.d., 1.8 μm; Waters, USA) at Majorbio Bio-Pharm Technology Co. Ltd. (Shanghai, China). The mobile phases consisted of 0.1% formic acid in water:acetonitrile (95:5, v/v) (solvent A) and 0.1% formic acid in acetonitrile:isopropanol:water (47.5:47.5, v/v) (solvent B). The flow rate was 0.40 mL/min and the column temperature was 40℃. The mass spectrometric data were collected using a Thermo UHPLC-Q Exactive HF-X Mass Spectrometer equipped with an electrospray ionization (ESI) source operating in positive mode and negative mode. The optimal conditions were set as followed: source temperature at 425℃; sheath gas flow rate at 50 arb; Aux gas flow rate at 13 arb; ion-spray voltage floating (ISVF) at -3500V in negative mode and 3500V in positive mode, respectively; Normalized collision energy, 20-40-60V rolling for MS/MS. Full MS resolution was 60000, and MS/MS resolution was 7500. Data acquisition was performed with the Data Dependent Acquisition (DDA) mode. The detection was carried out over a mass range of 70-1050 m/z.

**Data Analysis**

The pretreatment of LC/MS raw data was performed by Progenesis QI (Waters Corporation, Milford, USA) software, and a three-dimensional data matrix in CSV format was exported. The information in this three-dimensional matrix included: sample information, metabolite name and mass spectral response intensity. Internal standard peaks, as well as any known false positive peaks (including noise, column bleed, and derivatized reagent peaks), were removed from the data matrix, deredundant and peak pooled. At the same time, the metabolites were identified by searching database, and the main databases were the HMDB (http://www.hmdb.ca/), Metlin

(https://metlin.scripps.edu/) and Majorbio Database.

The data matrix obtained by searching database was uploaded to the Majorbio cloud platform (https://cloud.majorbio.com) for data analysis. Firstly, the data matrix was pre-processed, as follows: At least 80% of the metabolic features detected in any set of samples were retained. After filtering, for specific samples with metabolite levels below the lower limit of quantification, the minimum metabolite value was estimated and each metabolic signature was normalized to the sum. To reduce the errors caused by sample preparation and instrument instability, the response intensities of the sample mass spectrometry peaks were normalized using the sum normalization method, to obtain the normalized data matrix. Meanwhile, the variables of QC samples with relative standard deviation (RSD) > 30% were excluded and log10 logarithmicized, to obtain the final data matrix for subsequent analysis.

Then, the R package “ropls” was used to perform principal component analysis (PCA), least partial squares discriminant analysis (PLS-DA), orthogonal least partial squares discriminant analysis (OPLS-DA), and permutation tests evaluating the stability of the model. The metabolites with VIP>1, P-value<0.05 were determined as significantly different metabolites based on the variable importance in the projection (VIP) obtained by the PLS-DA or OPLS-DA model and the P-value generated by Welch’s t test.

Differential metabolites among two groups were mapped into their biochemical pathways through metabolic enrichment and pathway analysis based on KEGG database (http://www.genome.jp/kegg/). These metabolites could be classified according to the pathways they involved or the functions they performed. Enrichment analysis was used to analyze a group of metabolites in a function node whether appears or not. The principle was that the annotation analysis of a single metabolite develops into an annotation analysis of a group of metabolites. Python packages “scipy.stats” (https://docs.scipy.org/doc/scipy/) was used to perform enrichment analysis to obtain the most relevant biological pathways for experimental treatments.
